# Evidence of pre-descemetocele and stromal remodelling in post-DSAEK corneal melt

**DOI:** 10.1038/s44440-025-00008-2

**Published:** 2026-01-12

**Authors:** Alia Z. Al-Mousawi, Ahmed Hamdy Oreaba, Darren S. J. Ting

**Affiliations:** 1 Birmingham and Midland Eye Centre, Sandwell and West Birmingham NHS Trust, Birmingham, UK; 2 Academic Unit of Ophthalmology, Department of Inflammation and Ageing, School of Immunity, Infection, and Inflammation, College of Medicine and Health, University of Birmingham, Birmingham, UK; 3 Academic Ophthalmology, School of Medicine, University of Nottingham, Nottingham, UK; 4 Ophthalmology and Visual Sciences Academic Clinical Program, Duke-NUS Medical School, Singapore, Singapore; 5 Singapore Eye Research Institute, Singapore National Eye Centre, Singapore, Singapore

**Keywords:** Corneal diseases, Inflammation

Corneal melt is an uncommon but sight-threatening corneal emergency. It can be caused by various pathologies, including infection, inflammation, neurotrophic keratopathy, and chemical/thermal injury, amongst others [1]. Progressive corneal melt can lead to baring of Descemet membrane (DM), known as “descemetocele”, and subsequent corneal perforation [1]. Herein, we present a case of threatened corneal perforation in an eye with previous Descemet’s stripping automated endothelial keratoplasty (DSAEK) where the host DM was already removed, providing supportive evidence on the role of pre-Descemet’s layer/Dua’s layer (PDL) in corneal melt/perforation.

An 84-year-old lady, with a previously failed DSAEK, presented with a two-month gradual painless loss of left eye vision. At presentation, the left eye corrected-distance-visual-acuity (CDVA) was hand movement, and corneal sensation was completely absent. Slit-lamp examination/photography revealed a significant central corneal melt and a translucent bulging corneal structure resembling “‘descemetocele” **(**Fig. 1A**)**. Anterior segment optical coherence tomography (AS-OCT) similarly demonstrated a thin hyper-reflective, anteriorly herniated corneal structure resembling “descemetocele” (Fig. 1B). Seidel’s test was negative. The patient was treated for severe corneal melt secondary to active PCR-proven herpes simplex keratitis, neurotrophic keratopathy, and exposure keratopathy.

**Fig. 1:**
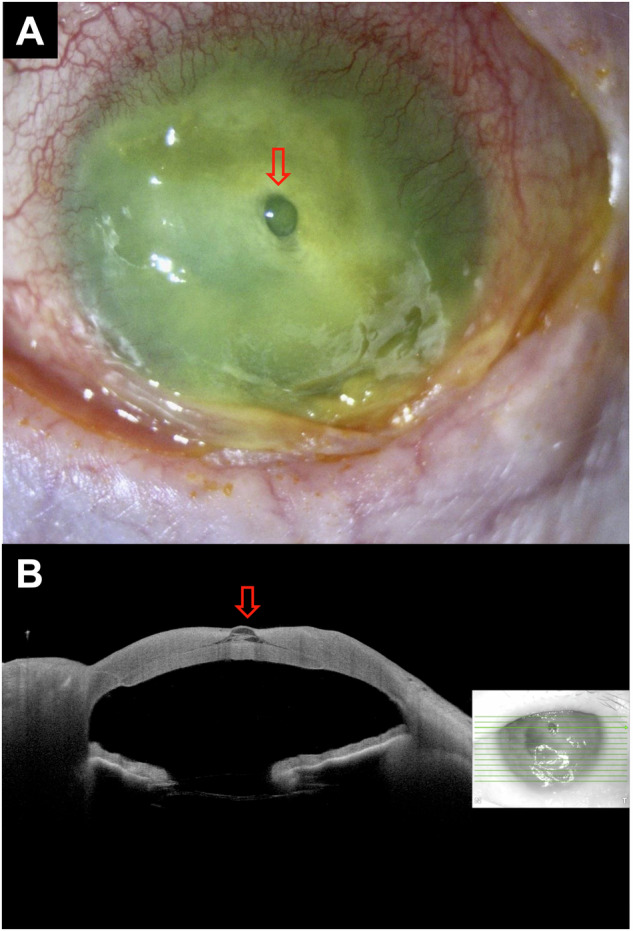
A case of “descemetocele” in an eye with prior Descemet stripping automated endothelial keratoplasty (DSAEK). **A** Slit-lamp photography demonstrating a central corneal melt with a “descemetocele” (red arrow) secondary to herpes simplex keratitis, neurotrophic keratopathy and exposure keratopathy. **B** Anterior segment optical coherence tomography (AS-OCT) showing a thin, hyper-reflective corneal tissue (red arrow) resembling a “pre-descemetocele”, with an underlying failed DSAEK graft.

Following 3 weeks of intensive medical management, significant stromal tissue migration/remodelling beneath the “pre-descemetocele” was noted, likely originating from the stromal tissue of DSAEK (Fig. 2A, B). Amniotic membrane grafting and tarsorrhaphy were subsequently performed to enable complete corneal healing (Fig. 2C).

**Fig. 2:**
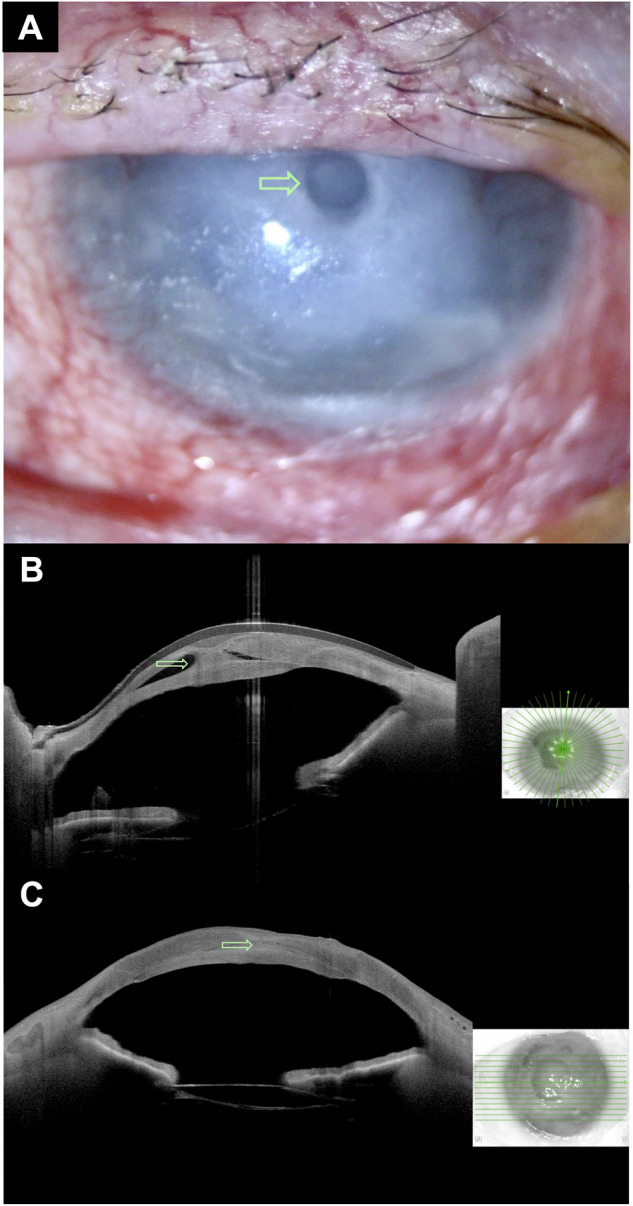
Healing of pre-descemetocele. **A** Slit-lamp photography demonstrating the healing of “pre-descemetocele” and thickening of the central cornea. Anterior segment optical coherence tomography (AS-OCT) showing significant stromal tissue remodelling beneath the “pre-descemetocele”, likely originating from DSAEK stromal tissues, at 3 weeks (**B**) and 6 months (**C**) after the initial presentation.

“Descemetocele” is a rare clinical entity characterised by anterior herniation of intact DM through an area of stromal thinning due to progressive corneal melt [1]. If left untreated, it may progress to corneal perforation with significant loss of vision. However, the discovery/characterisation of PDL has raised the question whether descemetocele is always truly DM-baring. Emerging evidence suggests that the posterior corneal biomechanical strength is conferred by PDL instead of DM, which usually ruptures with relatively low pressure as observed in DALK surgery (when a type-2 big-bubble is created) [2–4]. PDL has been described to contain predominantly collagen type I, collagen type IV, and a high density of elastin (which confers its tensile strength), with bursting pressures up to 700 mmHg [2, 4].

Based on AS-OCT findings, three main types of “descemetocele” have been described, including: Type 1 - herniation of DM with overlying PDL; Type 2 – herniation of DM alone; and Type 3 – herniation of DM with overlying PDL and a variable amount of corneal stroma [4]. This unique case further demonstrated that “descemetocele” may even present in the absence of DM, providing compelling evidence on the role of PDL in posterior corneal tensile strength. As the host DM was already removed during descemetorhexis in a previous DSAEK, the thin anterior herniated structure observed on AS-OCT likely represents the PDL (instead of the DM), which lends support to the use of the term “pre-descemetocele” over “descemetocele” in certain cases [3, 4].

Another point of interest highlighted by this case is the posterior tamponade provided by the DSAEK graft and the significant stromal remodelling noted beneath the “pre-descemetocele” during the healing process, likely originating from the DSAEK stromal tissue, which reinforces the role of tectonic mini-DSAEK in corneal perforations [5].

## Data Availability

All data supporting the findings of this work are available within the paper.
